# The Cytoplasmic Tail of Influenza A Virus Hemagglutinin and Membrane Lipid Composition Change the Mode of M1 Protein Association with the Lipid Bilayer

**DOI:** 10.3390/membranes11100772

**Published:** 2021-10-10

**Authors:** Larisa V. Kordyukova, Petr V. Konarev, Nataliya V. Fedorova, Eleonora V. Shtykova, Alexander L. Ksenofontov, Nikita A. Loshkarev, Lubov A. Dadinova, Tatyana A. Timofeeva, Sergei S. Abramchuk, Andrei V. Moiseenko, Lyudmila A. Baratova, Dmitri I. Svergun, Oleg V. Batishchev

**Affiliations:** 1 Belozersky Institute of Physico-Chemical Biology, Lomonosov Moscow State University, 119991 Moscow, Russia; kord@belozersky.msu.ru (L.V.K.); fedorova@belozersky.msu.ru (N.V.F.); ksenofon@belozersky.msu.ru (A.L.K.); baratova@belozersky.msu.ru (L.A.B.); 2 Shubnikov Institute of Crystallography, Federal Scientific Research Centre “Crystallography and Photonics” of Russian Academy of Sciences, 119333 Moscow, Russia; peter_konarev@mail.ru (P.V.K.); eleonora.shtykova@gmail.com (E.V.S.); lubovmsu@mail.ru (L.A.D.); 3 Laboratory of Bioelectrochemistry, Frumkin Institute of Physical Chemistry and Electrochemistry, Russian Academy of Sciences, 119991 Moscow, Russia; loshkarev.mipt.1994@gmail.com; 4 Laboratory of Physiology of Viruses, D. I. Ivanovsky Institute of Virology, FSBI N. F. Gamaleya NRCEM, Ministry of Health of Russian Federation, 123098 Moscow, Russia; timofeeva.tatyana@inbox.ru; 5 Department of Chemistry, Lomonosov Moscow State University, 119234 Moscow, Russia; abr@polly.phys.msu.ru; 6 Laboratory of Physical Chemistry of Polymers, A.N. Nesmeyanov Institute of Organoelement Compounds of Russian Academy of Sciences, 119991 Moscow, Russia; 7 Laboratory of Electron Microscopy, Department of Biology, Lomonosov Moscow State University, 119234 Moscow, Russia; postmoiseenko@gmail.com; 8 EMBL, Hamburg Unit, c/o DESY, 22607 Hamburg, Germany; svergun@embl-hamburg.de

**Keywords:** influenza a virus, lipoprotein envelope, M1 matrix protein, hemagglutinin, anchoring peptide, S-acylation, bromelain digestion, transmembrane domain (TMD), cytoplasmic tail (CT), unilamellar liposomes, small-angle X-ray scattering (SAXS), BILMIX program

## Abstract

Influenza A virus envelope contains lipid molecules of the host cell and three integral viral proteins: major hemagglutinin, neuraminidase, and minor M2 protein. Membrane-associated M1 matrix protein is thought to interact with the lipid bilayer and cytoplasmic domains of integral viral proteins to form infectious virus progeny. We used small-angle X-ray scattering (SAXS) and complementary techniques to analyze the interactions of different components of the viral envelope with M1 matrix protein. Small unilamellar liposomes composed of various mixtures of synthetic or “native” lipids extracted from Influenza A/Puerto Rico/8/34 (H1N1) virions as well as proteoliposomes built from the viral lipids and anchored peptides of integral viral proteins (mainly, hemagglutinin) were incubated with isolated M1 and measured using SAXS. The results imply that M1 interaction with phosphatidylserine leads to condensation of the lipid in the protein-contacting monolayer, thus resulting in formation of lipid tubules. This effect vanishes in the presence of the liquid-ordered (raft-forming) constituents (sphingomyelin and cholesterol) regardless of their proportion in the lipid bilayer. We also detected a specific role of the hemagglutinin anchoring peptides in ordering of viral lipid membrane into the raft-like one. These peptides stimulate the oligomerization of M1 on the membrane to form a viral scaffold for subsequent budding of the virion from the plasma membrane of the infected cell.

## 1. Introduction

Influenza A is an enveloped negative-strand RNA virus that belongs to the *Orthomyxoviridae* family [1]. It is one of the well-studied human pathogens, providing clues for understanding the pathogenesis of various enveloped viruses. However, the subtle mechanisms of pH-dependent penetration of the Influenza A virus genome into the host cell, as well as the assembly of the progeny virions, are still far from being understood. A lipoprotein envelope surrounding Influenza A virus nucleocapsid contains lipid molecules of the host cell membrane and three integral viral proteins: major antigen hemagglutinin (HA), 6–10 times less represented enzyme neuraminidase (NA), and minor protein M2, an ion channel [1]. The most abundant protein within the Influenza A virion, M1 matrix protein, forms a lattice under the lipid bilayer, which disintegrates at acidic pH, allowing viral ribonucleoprotein particles (RNPs) to enter the cytoplasm [2,3,4]. M1 comprises 252 amino acid residues (27.9 kDa, calculated MW for A/Puerto Rico/8/34 (H1N1) virus strain) and has the most conserved amino acid sequence among virus proteins [5]. The three-dimensional structure of the globular N-terminal fragment (residues 1–164) has been obtained by X-ray crystallography at pH 4.0 and pH 7.0 [2,6,7,8]. It contains four α-helices (H1 to H4, residues 2–67) in the N-terminal domain and four α-helices (H6 to H9, residues 91–158) in the M-(middle) domain. A coil containing a short helix H5 links the N- and M-domains. In contrast to the globular N-terminal domain of M1 comprising two-thirds of the full-length protein, a flexible C-terminal region (residues 165 to 252) as well as the full-sized protein have not been crystallized so far despite multiple attempts [6,7]. Recently, using small-angle X-ray scattering (SAXS), atomic force microscopy (AFM), and other approaches, we have provided insights into the mechanism of M1 scaffold formation and the major role of the flexible and disordered C-terminal domain in this pH-dependent process [3,9]. 

In recent years, M1 protein has been hypothesized to have either hydrophobic interactions with the inner monolayer of the viral lipid membrane [6,10] or electrostatic interactions with the phosphatidylserine (PS) [7,11] as the main partner in lipid membrane [12,13]. Consistent with these observations, the interaction between M1 and the membrane is mediated by a positively charged surface formed by helices 5, 6, and 8 according to recent achievements of cryo-electron tomography of Influenza A virion at the resolution of about 8 Å [14]. Positively charged residues of M1 that mediate its interactions with negatively charged lipid molecules are three arginines in positions 76–78 in H5, R101 and K104 in H6, and R134 in H8 [14]. Indirect evidence suggests that M1 should interact with cytoplasmic domains of all three integral viral glycoproteins, HA, NA, and M2, to form infectious virus progeny [15,16,17,18,19]. A strong interaction has been recently proposed for the M2-M1 protein pair recruiting M1 to the plasma membrane, possibly allowing its further interaction with other viral proteins [20]. However, the HA-M1 complexes have never been observed directly via biochemical or structural means. 

This study aimed to clarify the impact of various components of the viral envelope in lipid membrane re-structuring as a response to interaction with the M1 matrix protein. We suggest an approach based on small-angle X-ray scattering (SAXS) analysis of several types of unilamellar liposomes and proteoliposomes. We started from simple two-component negatively charged liposomes composed of synthetic lipids (DOPC/DOPS) to assess the effects of electrostatic M1-DOPS contacts on the lipid bilayer configuration. Secondly, we studied four-component (PS/POPC/SM/Chol) vesicles containing the lipid raft-forming constituents to test the impact of hydrophobic contacts and lipid rafts. Thirdly, we prepared “native” liposomes composed of a complex mixture of viral lipids extracted using the Folch method [21] from viral particles by the protocol described earlier [22]. Finally, we developed a new protocol to prepare proteoliposomes containing the same viral lipids together with the anchoring segments of integral viral proteins, mainly HA, as the amplest glycoprotein within the viral envelope. The HA anchoring segments remain in the viral envelope after bromelain digestion of the viral particles that are further subjected to the chloroform/methanol extraction. According to MALDI-TOF MS analysis we performed earlier, the major peptide extracted to chloroform phase from the A/Puerto Rico/8/34 (H1N1) bromelain digested virions is the 45 amino acid residues long C-terminal HA peptide with an amino acid sequence NH2-LESMGIYQILAIYSTVASSLVLLVSLGAISFWMCSNGSLQCRICI-COOH triply palmitoylated at three conserved cysteine residues [23,24,25], referred to here as LI45. Using the proteoliposome model system and isolated M1 protein, we may detect the interactions of M1 with Influenza A virus HA CT in the lipid environment. 

Analysis of asymmetric electron density profiles using the ATSAS program package suggests an essential impact of phosphatidylserine (PS), cholesterol (Chol), sphingomyelin (SM), and other lipids of the viral envelope on interactions with M1 matrix protein and following liposome membrane re-structuring. Of note, to the best of our knowledge, we detected for the first time an impact of viral anchoring peptides (mainly, the HA peptides) into the formation of lipoprotein nanodomains. Moreover, we suggested physico-chemical mechanisms underlying this process and its role in the processes of Influenza A virus assembly and budding. 

## 2. Materials and Methods

### 2.1. Synthetic Liposome Preparation

Synthetic liposomes were prepared from the following mixtures of synthetic lipids: (1) 10 mol.% of 1,2-dioleoyl-sn-glycero-3-phosphatidylserine (DOPS) plus 90 mol.% of 1,2-dioleoyl-sn-glycero-3-phosphatidylcholine (DOPC); (2) 30 mol.% of DOPS plus 70 mol.% of DOPC; (3) 30 mol.% of bovine brain extract of phosphatidylserines (bPS) plus 10 mol.% of 1-palmitoyl-2-oleoyl-sn- glycero-3-phosphatidylcholine (POPC) plus 40 mol.% of egg sphingomyelin (SM) plus 20 mol.% of cholesterol (Chol); (4) 20 mol.% of bPS plus 13.3 mol.% of POPC plus 33.3 mol.% of SM plus 33.3 mol.% of Chol. All lipids were purchased from Avanti Polar Lipids (AL, Alabaster, USA) and used without further purification. The lipid suspensions in the TNE buffer (100 mM NaCl, 10 mM Tris-HCl, 1 mM EDTA, pH 7.4) were extruded 19–21 times through a polycarbonate membrane with 100 nm pore size to gel unilamellar liposomes.

### 2.2. Virus Growth and Purification

Influenza A/Puerto Rico/8/34 (H1N1) (PR8) virus was propagated in 11-day embryonated chicken eggs for 48 h at 37 °C. The allantoic fluid was clarified by low-speed centrifugation (3500 rpm, 30 min), and virus particles were pelleted at 75,000× *g* for 1 h at 4 °C (Beckman JA-25 rotor, Beckman Coulter, Brea, CA, USA) and purified by centrifugation through 20% (vol/vol) sucrose in TNE buffer at 70,000× *g* for 1.5 h at 4 °C (SW-27 rotor, Beckman Coulter, Brea, CA, USA). The purity of particles was checked by SDS-PAGE (12% gel) according to Laemmli [26] in non-reducing conditions and Coomassie-staining. Viral protein was measured according to Lowry [27] or Peterson [28]. The UniProtKB database accession numbers are P03485 for the M1 protein and P03452 for the hemagglutinin of A/Puerto Rico/8/34 (H1N1) virus strain.

### 2.3. Preparation of the Subviral Particles 

Subviral particles were prepared by digestion of the purified virions in the absence of reducing agents with bromelain as described earlier [23,29]. Influenza A virions were incubated with bromelain (B5144, Sigma-Aldrich, St. Louis, MI, USA) in TE-buffer (100 mM Tris-HCl, 1 mM EDTA, pH 7.2) for 16 h at 35 °C using virus protein to enzyme protein ratios of 4 to 1 (0.25 mg/mL bromelain). The reaction was stopped by adding the protease inhibitor E-64 (N-(trans-Epoxysuccinyl)-L-leucine 4-guanidinobutylamide) to a final concentration of 10 µM. The subviral particles were pelleted through a 20% sucrose cushion in TNE buffer at 100,000× *g* for 1.5 h at 4 °C (Beckman SW-50.1 rotor, Beckman Coulter, Brea, CA, USA) to remove enzymes, and the virus pellet was resuspended in a small volume of TNE buffer. The HA removal from virions after bromelain digestion was confirmed using electron microscopy and SDS-PAGE electrophoretic analysis [23,29]. 

### 2.4. Preparation of Native Viral Liposomes and Proteoliposomes with HA LI45 Peptides

**Figure 1:**
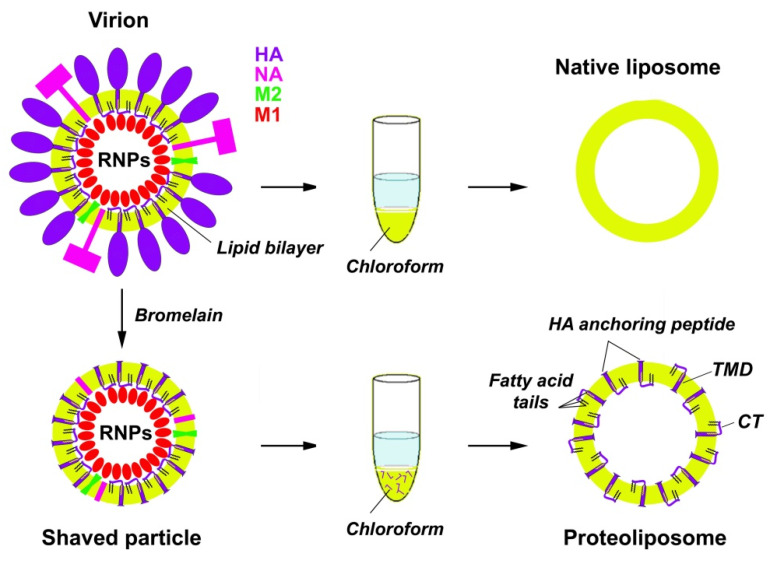
A scheme demonstrating protocols for preparation of native liposomes and proteoliposomes from purified PR8 virions and subviral particles, respectively. Influenza A virus structural proteins are designated as HA—hemagglutinin, NA—neuraminidase, M2, M1. The virus nucleocapsid composed of eight single-stranded negative-sense RNA molecules complexed with nucleoprotein and RNA polymerase proteins is designated as RNPs (ribonucleoprotein particles) for simplicity. Within the proteoliposomes, the HA LI45 peptides are designated as violet patterns. They possess the transmembrane domain (TMD), cytoplasmic tail (CT) regions, and S-acylation modification (three fatty acid tails bound via thioester bonds) anchoring the CT region to the lipid bilayer as indicated.

“Native” liposomes composed of viral lipids and proteoliposomes composed of viral lipids and containing the C-terminal HA anchoring peptides (LI45) were prepared in a similar way using the protocols of [22] with some modifications. The protocols for preparation of native liposomes and proteoliposomes are schematically depicted in Figure 1. 

Suspensions of purified PR8 virions (0.5–1.0 mL; ~4 mg viral protein/mL) or subviral particles (0.5–1.0 mL; ~2 mg viral protein/mL) prepared by bromelain digestion of the same virus in TNE buffer were mixed with 3 vol of the chloroform-methanol mixture (2:1, v/v) according to the modified Folch method [21], vortexed for 1–2 min, agitated in a shaker at room temperature for 30 min, and centrifuged at 1000× *g* for 5 min (Beckman Coulter Microfuge 18, Beckman Coulter, Brea, CA, USA). The upper (water/methanol) phase and interphase were discounted, while the low (chloroform) phase was collected and stored at −20 °C until use. Using a vacuum evaporator, the lipid film was obtained in the round-bottom flask, then rehydrated in TNE buffer, pH 7.2, at the temperature of ~50 °C (above the phase transition temperature of lipids). The flask was vortexed for ~10 min to detach the lipid film from the glass surface, and the obtained suspension of lipids was extruded 19–21 times through 1–2 polycarbonate membranes with 100 nm pore size. 

### 2.5. Isolation of M1 Protein 

The M1 protein was isolated from purified influenza A/Puerto Rico/8/34 virions at acid pH using the protocol of Zhirnov [30]. The identity and purity of the protein was proved using Laemmli SDS-PAGE electrophoresis [26] followed by Coomassie or silver staining and trypsin in-gel hydrolysis/MALDI-TOF mass [31]. The M1 protein solution was dialyzed using Bio-Beads (Bio-Rad, Hercules, CA, USA) in 100 mM NaCl/20 mM MES buffer (pH 4.7) and adjusted to pH 6.8 prior to incubation with liposomes.

### 2.6. Dynamic Light Scattering (DLS) Analysis

For the DLS measurements, the samples were diluted with TNE buffer to a protein concentration of 0.1–0.5 mg/mL and transferred to a polystyrene cuvette (10 mm). The volume of analyzed preparations was 0.5–1 mL. Light-scattering experiments were conducted at 22 °C with a Zetasizer Nano ZS instrument (Malvern Panalytical, Malvern, UK) at a laser wavelength of 633 nm. Seven measurements were carried out, each comprising 20 runs. Measured data were processed using DISPERSION TECHNOLOGY Software version 5.10. 

### 2.7. Negative-Stain Transmission Electron Microscopy (EM)

Liposome preparations were deposited on formvar–carbon-coated grids (TED Pella, Redding, CA, USA) and incubated for 2 min. After that, excess solution was removed, and the samples were stained for 20 s with a 2% water solution of phosphotungstic acid (Sigma-Aldrich, St. Louis, MI, USA), pH 7.0. Samples were viewed with a transmission electron microscope LEO 912 AB OMEGA (Carl Zeiss, Jena, Germany) or with the JEM-2100 200 kV electron microscope (JEOL, Tokyo, Japan), equipped with the LaB6 electron source. The images were taken with Gatan Ultrascan 1000XP 2k CCD camera (Gatan, Warrendale, PA, USA) at parallel illumination conditions and defocus between −1 and −2 μm. 

### 2.8. Small-Angle X-ray Scattering

SAXS experiments were performed at the EMBL P12 beamline, storage ring Petra-3, DESY, Hamburg, Germany [32]. Solutions of synthetic liposomes, native liposomes, and proteoliposomes containing the HA peptide LI45 were measured without and with loaded M1 protein at 283 K at the concentration range 1.0–3.0 mg/mL in the 100 mM NaCl, 50 mM MES buffer. Different molar ratios of liposomes to M1 protein were used: (67:1, 30:1, 20:1, 15:1, 10:5, 7.5:1, 5:1) for synthetic liposomes, (80:1, 40:1, 20:1, 10:1, 4:1, 2.6:1) for native liposomes, (5:1, 4:1, 2.6:1, 1:1) for liposomes containing LI45 peptide. The samples were measured immediately upon mixing and after 2 h incubation time. Five different compositions of synthetic liposomes were studied: 10% DOPS + 90% DOPC; 30% DOPS + 70% DOPC; 20% bPS + 13.3% POPC + 33.3% SM + 33.3% Chol; 30% bPS + 10% POPC + 40% SM + 20% Chol. The data at P12 beamline were recorded using a Pilatus 6 M detector (DECTRIS, Switzerland) with 20 × 0.05 seconds exposure time, at sample-detector distance 3.00 m and wavelength 1.24 Å covering the momentum transfer range 0.02 nm^−1^ < *s* < 7.0 nm^−1^ (with *s = 4πsinθ/λ*, where 2*θ* is the scattering angle and *λ* is the wavelength). No measurable radiation damage was detected by comparison of successive time frames. 

The data were processed with PRIMUS [33] from the ATSAS package [34] using standard procedures. The data were normalized by the transmitted intensity, and the buffer contribution was subtracted. 

### 2.9. Modeling the Electron Density Distribution of the Phospholipid Bilayer

The phospholipid bilayer electron density profiles of liposomes and the vesicle size distributions were restored using the program BILMIX [35]. For unloaded (control) liposomes, the symmetric electron density profile composed of three Gaussian functions was employed (representing the hydrophilic phospholipid polar headgroups and the hydrophobic hydrocarbon chains), whereas for loaded liposomes, the asymmetry of the bilayer profile (due to interactions with M1 protein) was introduced by additional Gaussian function.

The corresponding structural parameters (mean size, polydispersity, etc.) were optimized to provide the best fit to the experimental data, minimizing the discrepancy value χ^2^ calculated in the following way:

(1) $$\chi^{2}=\frac{1}{N-1}\underset{j}{\sum }{\left[\frac{I_{exp}\left(s_{j}\right)-cI_{calc}\left(s_{j}\right)}{\sigma \left(s_{j}\right)}\right]}^{2}$$

where N is the number of experimental points, *I_exp_(s_j_)* and *σ(s_j_*) are the experimental intensities and their error estimates, *I_calc_(s_j_)* is the calculated intensity from the liposome-M1 mixture model, and c is the scaling coefficient.

## 3. Results and Discussion

### 3.1. Synthetic Liposomes

To find an impact of negatively charged lipids and lipids of liquid-ordered domains (rafts) on the formation of M1-lipid complexes, two types of synthetic liposomes were prepared: first, the vesicles composed of DOPS/DOPC mixture, and second, those composed of PS/POPC/SM/Chol mixture, with various proportions of individual lipids. 

#### 3.1.1. Electron Microscopy (EM), Dynamic Light Scattering (DLS), and Small-Angle X-ray Scattering (SAXS) Analysis of Size Distributions of Synthetic Liposomes

**Figure 2:**
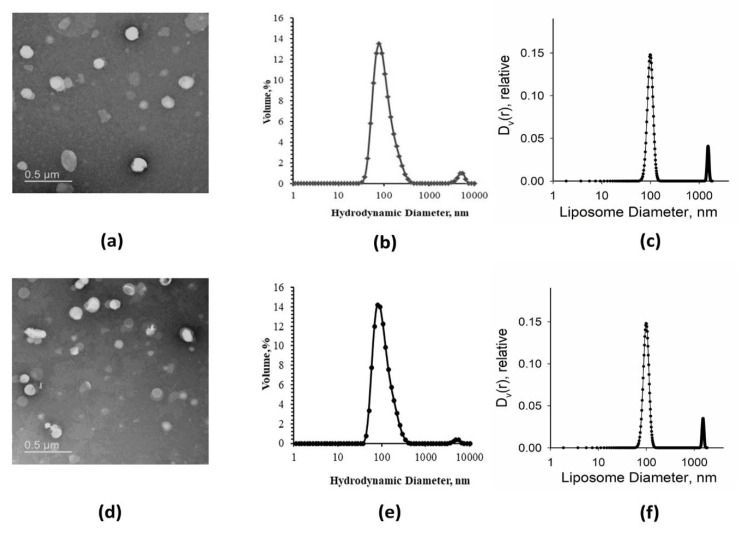
Size estimation of synthetic liposomes. Represented are EM images (**a**,**d**), DLS analysis data (**b**,**e**), and SAXS analysis data (**c,f**) obtained for synthetic liposomes composed of 10 mol.% of DOPS + 90 mol.% of DOPC mixture (**a**–**c**) or 30 mol.% of DOPS + 70 mol.% of DOPC mixture (**d**–**f**).

For the lipid vesicles composed of DOPS/DOPC, individual vesicles prevailed with their diameter varying from ~80 to 200 nm (Figure 2a,d). We applied the DLS analysis to assess the average hydrodynamic diameter of synthetic liposomes. It revealed a major symmetric peak with the maximum at 100 nm and a tiny peak of large size aggregates (Figure 2b,e). Similar size distributions were also obtained from SAXS data analysis (Figure 2c,f).

Generally speaking, liposomes obtained via extrusion through 100 nm pore diameter in polycarbonate membranes had different sizes [36,37,38]. As seen from EM images (Figure 2a,d), they were roughly from 50 to 300 nm in diameter. However, the main peak from DLS measurements corresponded to 100 nm diameter, which was the dominant size of the liposomes.

#### 3.1.2. SAXS Analysis of the Electron Density Profiles of Synthetic Liposomes

**Figure 3:**
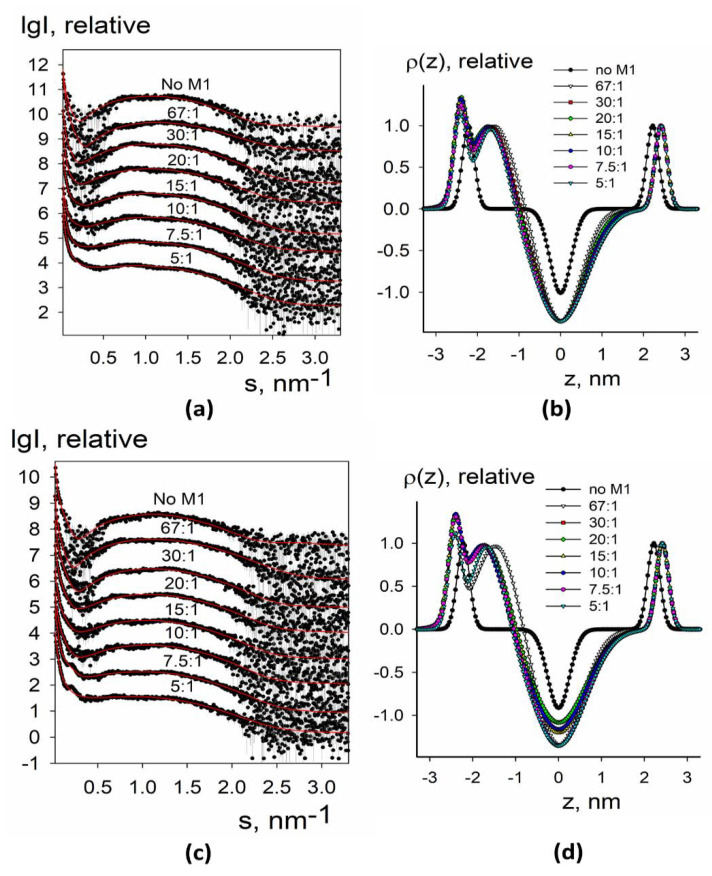
SAXS and BILMIX analysis of synthetic liposomes composed of 10 mol.% DOPS + 90% mol. DOPC (**a**,**b**) or 30 mol.% DOPS + 70 mol.% DOPC (**c,d**) lipid mixtures loaded with M1 protein. (**a**,**c**) Experimental small-angle scattering data are represented by dots with error bars. The best fits of the SAXS curves obtained by BILMIX program are shown by solid red lines. The curves are shifted by one logarithmic order for better clarity; (**b**,**d**) the restored electron density profiles of the lipid bilayer before (black curve) and after (color curves) loading with M1. The molar lipid/M1 ratios are the following: no M1, 67:1, 30:1, 20:1, 15:1, 10:1, 7.5:1, 5:1.

**Figure 4:**
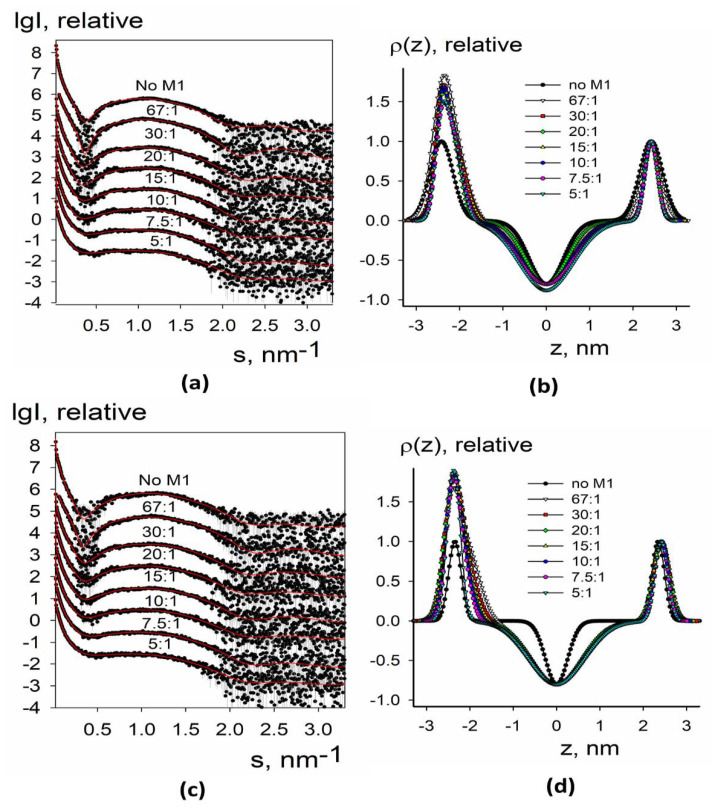
SAXS and BILMIX analysis of synthetic liposomes composed of 30 mol.% bPS + 10 mol.% POPC + 40 mol.% SM + 20 mol.% Chol (**a**,**b**) or 20 mol.% bPS + 13.3 mol.% POPC + 33.3 mol.% SM + 33.3 mol.% (**c**,**d**) lipid mixtures loaded with M1 protein. (**a**,**c**) Experimental small-angle scattering data are represented by dots with error bars. The best fits of the SAXS curves obtained by BILMIX program are shown by solid red lines. The curves are shifted by one logarithmic order for better clarity; (**b**,**d**) the restored electron density profiles of the lipid bilayer before (black curve) and after (color curves) loading with M1. The molar lipid/M1 ratios are the following: no M1, 67:1, 30:1, 20:1, 15:1, 10:1, 7.5:1, 5:1.

Experimental small-angle X-ray scattering patterns obtained for synthetic liposomes without and with added M1 protein are presented in Figure 3a,c and Figure 4a,c. The data from protein-free liposomes contained a wide maximum in the middle part of the angular range that is typical for the bilayer lipid vesicles [39]. At the same time, at low angles, there was a fast upturn of the scattering intensity that suggested the presence of large particles (with diameters of up to 1000–1500 nm) and vesicle size polydispersity. Such characteristic features of the curves (upturn at low angles and wide peak in the middle part) were present for all samples and could be taken into account within separated form factor (SFF) approximation [40], where the scattering signal is represented as a product of the form factor from large spherical shells (providing information about vesicle size) and the form factor from the flat lipid bilayer (describing the electron density profile across the bilayer). The algorithm implemented in the program BILMIX [35] takes into account the size polydispersity of the vesicles and permits one to model both symmetric and asymmetric electron density profiles. We employed the above algorithm for modeling the experimental small-angle X-ray scattering data (see Materials and Methods for details).

We were able to neatly fit the experimental data using this approach (Figure 3a,c and Figure 4a,c, solid curves). At all conditions, the systems remained quite polydisperse and consisted of several fractions with the characteristic radii of 40–50 nm and 1000–1500 nm, similar to DLS size estimations (see Figure 2). The restored electron density profiles are shown in Figure 3b,d and Figure 4b,d. Black curves in Figure 3b,d and Figure 4b,d represent electron density profiles for protein-free liposomes. These curves were symmetrical with the minimum at the intermonolayer region of the lipid bilayer and two maxima at the regions of lipid polar headgroups. Distance between these maxima was about 4 nm, that is, the typical thickness of the lipid bilayer [41]. 

For two-component synthetic liposomes, the addition of the M1 decreased the electron density in the inter-monolayer region, while widening the minimum. The right maximum moved further to the right, meaning the increase in the effective thickness of the inner monolayer, and the left maximum split into two sub-peaks, one of which moved to the central region of the membrane while widening, and the other of which moved to the left and increased in intensity. 

This behavior can be explained as follows: the splitting of the peak corresponds to the appearance of a protein, and the left-most peak with the increased intensity corresponds to the M1 protein adsorbed at the liposome. Protein adsorption leads to the condensation of lipids underneath, i.e., their area per the headgroup decreases. The protein condenses anionic lipids, i.e., DOPS, as has been shown earlier [11]. Therefore, for the composition of 30 mol.% DOPS + 70 mol.% DOPC, the effect is stronger than for 10 mol.% DOPS + 90 mol.% DOPC (compare Figure 3b,d). As a result of this condensation, the dipoles of the polar lipid headgroups straighten, and therefore the electron density peak of the polar headgroups for this monolayer becomes wider. In this case, an imbalance in the area of monolayers occurs, leading to the bending of the membrane inside the liposome at the sites of protein adsorption. We may suggest that it leads to the formation of tubular invaginations inside lipid vesicles, similar to the structures induced by the M1 protein of the Influenza C virus [42]. Such local bends result in an effective reduction in the thickness of the outer (protein-contacting) monolayer and an increase in the thickness of the inner. 

We further tried liposomes with more complex lipid composition closely describing the lipid raft composition of the membrane [43]. Two important lipid raft constituents, cholesterol and sphingomyelin, were introduced into the liposomes in different mole portions. The results are represented in Figure 4.

For four-component synthetic liposomes, the central minimum was much lower, which reflects the increased order of the lipid tails because this lipid composition provides the formation of large liquid-ordered lipid domains [43]. In this system, we did not observe a separated protein-associated peak; however, the intensity of the left maximum increased. Additionally, the right peak did not shift from its position for protein-free liposomes. We may suggest that the presence of cholesterol and sphingomyelin stabilizes the lipid bilayer and prevents the M1-induced condensation of charged lipids. Every membrane deformation depends on membrane rigidity. In terms of theory of elasticity of liquid crystals adopted to lipid membranes, the energy of deformation of lipid bilayer depends on elastic moduli of the membrane [44]. Formation of membrane protrusions is mostly regulated by the bending modulus of lipid bilayer, which is much greater for the liquid-ordered membrane [45]. As a result, the lipid bilayer invaginations inside the inner vesicle volume are not formed in raft-forming lipid mixtures. Notably, we did not detect any difference between 30 mol.% bPS + 10 mol.% POPC + 40 mol.% SM + 20 mol.% Chol and 20 mol.% bPS + 13.3 mol.% POPC + 33.3 mol.% SM + 33.3 mol.%. This fact suggests that the change of SM/Chol ratio does not affect M1 adsorption and its ability to produce membrane protrusions. 

### 3.2. Native Liposomes and Proteoliposomes 

We prepared native liposomes composed of a complex mixture of lipids from the PR8 viral envelope and proteoliposomes composed of the viral lipids and the anchoring fragments of integral viral proteins, mainly, the HA C-terminal peptides (LI45) inserted into the lipid bilayer. According to [22], four major phospholipids are present in the lipid mixture extracted from the PR8 virus (in mol.%): sphingomyelin (22.1), phosphatidylserine (22.2), phosphatidylethanolamine (33.3), and phosphatidylcholine (15.1). The ratio of cholesterol over total lipids was about 0.43. 

The preparation of proteoliposomes includes an additional stage, namely, the bromelain digestion of the purified virions, which removes the ectodomains of the surface glycoproteins. The anchoring segments of HA were extracted into the chloroform phase together with viral lipids because they were very hydrophobic: first, because of the hydrophobic nature of TMD, second, because of modification of CT with three palmitate residues (C16:0). We sometimes detected the NA TMD-containing peptides as minor peaks in the mass spectra of chloroform phase obtained for subviral particles of some Influenza A virus strains, but not PR8, and we never detected the M2-derived peptides (albeit they are singly palmitoylated at a cysteine residue and, thus, rather hydrophobic), probably because of the inefficiency of bromelain digestion of those proteins and/or their low copy representation [29].

In contrast to the situation with native virions, where the CT region is located inside the virus particle and might interact with an M1 protein sheet, in our model proteoliposome system, two orientations of the HA LI45 peptide are possible, both CT inside the vesicles (as within the virions) and also outside them (see Figure 1). In the latter case, it could interact with the M1 protein adsorbed from the solution. Of note, three covalently bound fatty acid residues should attach the CT to the lipid bilayer, thus positioning it along the membrane [46].

#### 3.2.1. EM and DLS Analysis of Native Liposomes and Proteoliposomes

**Figure 5:**
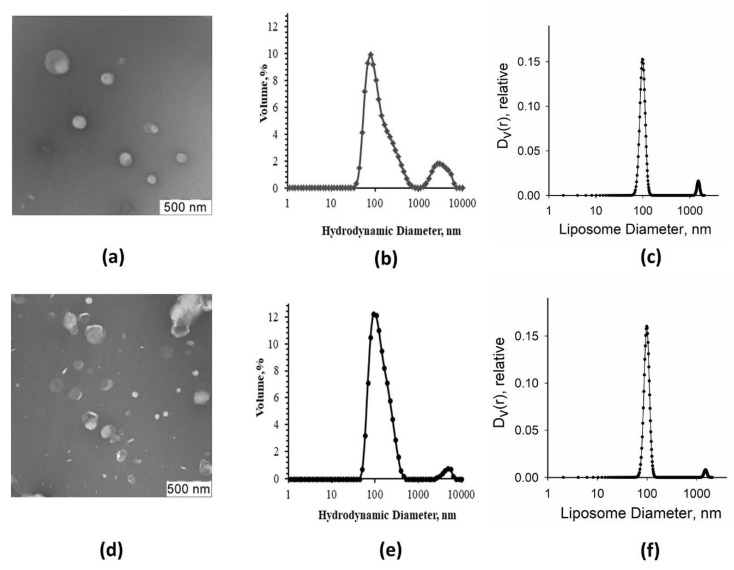
Size estimation of native liposomes and proteoliposomes. Represented are the EM images (**a**,**c**), the DLS analysis (**b**,**e**), and the SAXS analysis results (**c**,**f**) obtained for native liposomes (**a**–**c**) or proteoliposomes (**d**–**f**).

EM images of native liposomes and proteoliposomes are shown in Figure 5a,d. After extrusion through membranes with a pore size of 100 nm, the preparations contained individual liposomes of quite different sizes (roughly, 50–300 nm in diameter). The DLS and SAXS analyses revealed a major peak of vesicles with a diameter around 100 nm, and a smaller peak of liposome aggregates in both native liposome and proteoliposome preparations (Figure 5b,c,e,f). 

#### 3.2.2. SAXS Analysis of Electron Density Profiles of Native Liposomes and Proteoliposomes

**Figure 6:**
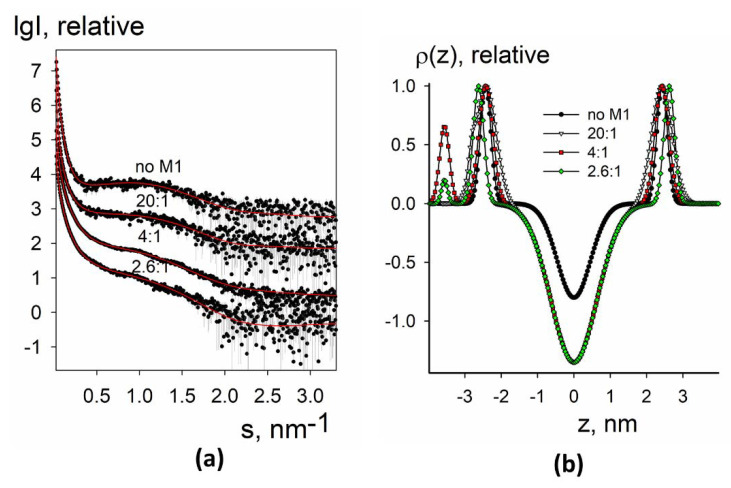
SAXS and BILMIX analysis of native liposomes composed of lipids extracted from A/Puerto Rico/8/34 (H1N1) virus envelope loaded with M1 protein. (**a**) Experimental are shown by dots with error bars, the fits obtained by BILMIX are shown as red curves. The curves are shifted by one logarithmic order for better clarity; (**b**) the restored electron density profiles of the lipid bilayer before (black curve) and after (color curves) loading with M1. The molar lipid:M1 ratios are the following: no M1, 20:1, 4:1, 2.6:1.

**Figure 7:**
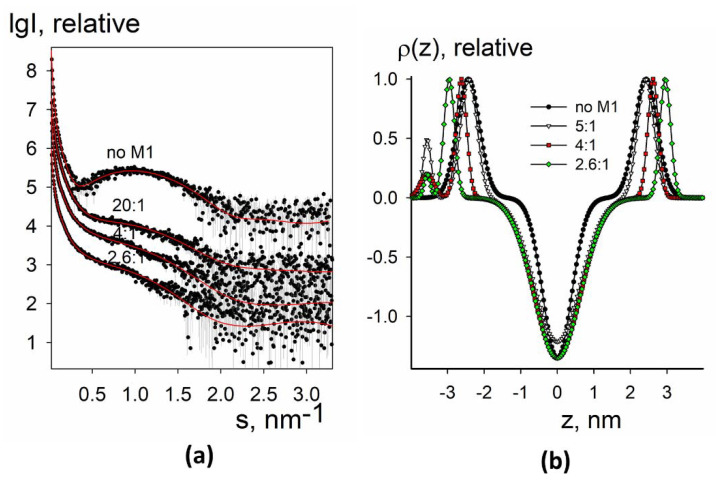
SAXS and BILMIX analysis of proteoliposomes composed of lipids from A/Puerto Rico/8/34 (H1N1) virus envelope together with the HA LI45 peptides loaded with M1 protein. (**a**) Experimental curves are shown by dots with error bars; the fits obtained by BILMIX are shown as red curves. The curves are shifted by one logarithmic order for better clarity; (**b**) the restored electron density profiles of the lipid bilayer before (black curve) and after (color curves) loading with M1. The molar lipid:M1 ratios are the following: no M1, 5:1, 4:1, 2.6:1.

Experimental small-angle X-ray scattering patterns from liposomes without and with the M1 protein are presented in Figure 6a and Figure 7a, together with the fitted data. The restored electron density profiles are shown in Figure 6b and Figure 7b. 

In the case of native liposomes, the electron density profile was closer to two-component synthetic liposomes rather than to four component raft-forming ones in some parameters (see below) (Figure 6b). This is an interesting fact because it is commonly believed that the lipid membrane of the Influenza A virus is mainly in a liquid-ordered state [47]. For liposomes containing LI45 peptide, electron density at the intermonolayer surface was minimal (Figure 7b), as for four-component synthetic liposomes. This result indicates that only the presence of LI45 peptide with three palmitates makes lipid tails more ordered, making viral membrane raft-like. 

For both native liposomes and liposomes containing LI45 peptide, we observed the appearance of a protein-associated peak on the electron density profile of the lipid bilayer (Figure 6b and Figure 7b), as for the two-component negatively charged synthetic liposomes with the adsorbed M1 protein. However, here it was separated from the second (lipid-associated) peak and had less intensity compared to the two-component synthetic liposomes. This observation suggests that in the case of native liposomes with or without LI45 peptide, M1 introduces its helices into the lipid bilayer. This is close to the results of cryo-electron microscopy of intact virions, which indicate the presence of some gap between the M1 layer and lipid membrane of the virus [48]. For the native liposomes, we also observed the drop of the electron density at the intermonolayer surface (Figure 6b) upon adsorption of the M1 protein, while for the liposomes containing LI45 peptide, this density was minimal already for liposomes free from M1 (Figure 7b). The bilayer of the native liposomes again demonstrated somewhat similar behavior to the two-component synthetic liposomes: the peak of the M1 interacting monolayer moved left, while the peak of the inner monolayer moved slightly right. However, shifting of the second sub-peak closer to the bilayer center (as it was in the case of negatively charged two-component liposomes) was not observed in the native liposomes. Thus, membrane tubular invaginations are probably specific for synthetic mixtures rather than for the complex mixtures of viral lipids, which exist in a more ordered state.

Moreover, one can see that the inner monolayer of the liposome was thickening upon adsorption of M1 because the corresponding peak moved to the right after the addition of the protein in solution. Notably, this effect was more pronounced in the proteoliposomes compared to native liposomes: the whole width of their lipid bilayer increased by 2 nm (1 nm at each side). This shows the additional impact of the HA anchoring segments on the formation of thick lipid-protein nanodomains. Recently, based on the results of studying the secondary structure of a series of synthetic peptides corresponding to the HA CT sequence (H1 subtype), we proposed a model of this region containing an antiparallel β-structure with a turnaround and invariant glycine residue [49]. The height/depth of the β-hairpin is ~ 1 nm, which is consistent with the widening of the lipid bilayer of proteoliposomes containing such HA peptide configuration for about 1 nm at each side after loading them with M1. We assume that LI45 peptides stimulate the oligomerization of M1 protein and the formation of LI45-M1 complexes, which in turn enhance the self-association of M1 protein and the formation of a layer of M1 molecules on the surface of the lipid bilayer. 

Thus, in the context of Influenza A virus assembly and budding, we can propose a specific role of the HA CTs probably serving as “seeds” for the M1 oligomerization; these contacts may stimulate the formation of a layer of M1 molecules on the surface of the lipid bilayer, facilitating the process of virus particle assembly. 

## Data Availability

The collected SAXS data and the restored electron density profiles have been deposited and are available at Small-Angle Scattering Biological Data Bank (SASBDB) [50], under the following codes: SASDMF5—synthetic liposomes composed of 10% DOPS + 90% DOPC loaded with M1 protein; SASDMG5—synthetic liposomes composed of 30% DOPS + 70% DOPC loaded with M1 protein; SASDMH5—synthetic liposomes composed of 30% bPS + 10% POPC + 40% SM + 20% Chol loaded with M1 protein; SASDMJ5—synthetic liposomes composed of 20% bPS + 13.3% POPC + 33.3% SM + 33.3% loaded with M1 protein; SASDMK5—native liposomes composed of lipids extracted from A/Puerto Rico/8/34 (H1N1) virus envelope loaded with M1 protein; SASDML5—proteoliposomes composed of lipids from A/Puerto Rico/8/34 (H1N1) virus envelope together with the HA LI45 peptides loaded with M1 protein.
